# Supplementary material for: Evaluating the impact of DREAMS on HIV incidence among adolescent girls and young women: A population-based cohort study in Kenya and South Africa
Source: PLoS Med. 2021 Oct 25;18(10):e1003837. doi: 10.1371/journal.pmed.1003837 (PMC8880902; doi:10.1371/journal.pmed.1003837)
Supplement: S1 Table — (DOCX) [file pmed.1003837.s002.docx]

**S1 Table.** Mean age (SD) of HIV-negative AGYW who are repeat-testers (in the HIV incidence cohort) and those who do not have a repeat test in uMkhanyakude

| Year | Eligible for entry to cohort^1^ | Repeat testers (in cohort) ^2^ | Mean age (SD) (years) | Non-repeat testers^3^ | Mean age (SD) (years) |
| --- | --- | --- | --- | --- | --- |
| 2006 | 3246 | 2700 | 19.2 (2.48) | 546 | 18.7 (2.47) |
| 2007 | 3268 | 2595 | 19.6 (2.51) | 673 | 18.9 (2.51) |
| 2008 | 3103 | 2569 | 19.8 (2.53) | 534 | 19.0 (2.51) |
| 2009 | 2763 | 2232 | 20.2 (2.36) | 531 | 19.5 (2.36) |
| 2010 | 3018 | 2383 | 19.8 (2.61) | 635 | 19.3 (2.55) |
| 2011 | 2791 | 2214 | 19.9 (2.50) | 577 | 19.4 (2.48) |
| 2012 | 2757 | 2070 | 20.2 (2.51) | 687 | 19.4 (2.50) |
| 2013 | 3000 | 2248 | 20.0 (2.69) | 752 | 19.1 (2.61) |
| 2014 | 2987 | 2264 | 20.1 (2.61) | 723 | 19.1 (2.59) |
| 2015 | 3087 | 2426 | 19.7 (2.59) | 661 | 18.9 (2.54) |
| 2016 | 3395 | 2525 | 19.3 (2.82) | 870 | 18.8 (2.58) |
| 2017 | 3180 | 2194 | 19.6 (2.68) | 986 | 19.0 (2.48) |
| 2018 | 3153 | 1809 | 19.1 (2.91) | 1344 | 19.1 (2.42) |

^1^Number of AGYW who first tested HIV negative when aged <25 years, are eligible for entry into the HIV incidence cohort, and are still aged <25 years.

^2^Number of eligible HIV negative AGYW who had a repeat test, and contributed person time to the HIV incidence analysis during each calendar period.

^3^Number of eligible HIV negative AGYW who did not have a repeat test, so did not contribute person-time to the HIV incidence analysis.
