# Supplementary material for: Evaluating the impact of DREAMS on HIV incidence among adolescent girls and young women: A population-based cohort study in Kenya and South Africa
Source: PLoS Med. 2021 Oct 25;18(10):e1003837. doi: 10.1371/journal.pmed.1003837 (PMC8880902; doi:10.1371/journal.pmed.1003837)
Supplement: S2 Table — (DOCX) [file pmed.1003837.s003.docx]

**S2 Table.**  Incidence of HIV infection among AGYW in Gem, by age and DREAMS implementation period: *sensitivity analysis without residency gaps*

| **Age group** | **Calendar period** | | **New HIV infections** | | **Person-years** | | **Incidence rate / 100 person-years** | | | **Age-adjusted rate ratio (95% CI) 1** | | |  |  |
| --- | --- | --- | --- | --- | --- | --- | --- | --- | --- | --- | --- | --- | --- | --- |
| *Comparison of 2 calendar periods (a priori analysis)* | | | | | | | | | | | | | |  |
| 15-19 years | | 2010-2015 | | 28 | | 6923 | | 0.40 (2.79-5.86) | | | 1 | | |  |
|  | | 2016-2019 | | 14 | | 5335 | | 0.26 (1.55-4.43) | | | 0.65 (0.34-1.23) | | |  |
| 20-24 years | | 2010-2015 | | 38 | | 4657 | | 0.82 (5.94-11.21) | | | 1 | | |  |
|  | | 2016-2019 | | 19 | | 3121 | | 0.61 (3.89-9.53) | | | 0.75 (0.43-1.29) | | |  |
| 15-24 years | | 2010-2015 | | 66 | | 11580 | | 0.57 (4.48-7.25) | | | 1 | | |  |
|  | | 2016-2019 | | 33 | | 8456 | | 0.39 (2.78-5.49) | | | 0.68 (0.45-1.04) | | |  |
| *Comparison of 3 calendar periods (a posteriori analysis)* | | | | | | | | | | | | | |  |
| 15-19 years | 2010-2012 | | 16 | | 2452 | | | | 0.65 (3.99-10.65) | | | 2.43 (1.15-5.14) | | |
|  | 2013-2015 | | 12 | | 4470 | | | | 0.26 (1.52-4.73) | | | 1 | | |
|  | 2016-2019 | | 14 | | 5335 | | | | 0.26 (1.55-4.43) | | | 0.98 (0.45-2.11) | | |
| 20-24 years | 2010-2012 | | 15 | | 1834 | | | | 0.82 (4.93-13.55) | | | 1.00 (0.52-1.92) | | |
|  | 2013-2015 | | 23 | | 2823 | | | | 0.82 (5.41-12.27) | | | 1 | | |
|  | 2016-2019 | | 19 | | 3121 | | | | 0.61 (3.89-9.53) | | | 0.75 (0.41-1.37) | | |
| 15-24 years | 2010-2012 | | 31 | | 4287 | | | | 0.72 (5.08-10.28) | | | 1.51 (0.93-2.44) | | |
|  | 2013-2015 | | 35 | | 7293 | | | | 0.48 (3.45-6.69) | | | 1 | | |
|  | 2016-2019 | | 33 | | 8456 | | | | 0.39 (2.78-5.49) | | | 0.81 (0.51-1.31) | | |
