# Supplementary material for: Evaluating the impact of DREAMS on HIV incidence among adolescent girls and young women: A population-based cohort study in Kenya and South Africa
Source: PLoS Med. 2021 Oct 25;18(10):e1003837. doi: 10.1371/journal.pmed.1003837 (PMC8880902; doi:10.1371/journal.pmed.1003837)
Supplement: S3 Table — (DOCX) [file pmed.1003837.s004.docx]

**S3 Table**. HIV incidence estimates in young men aged 20–29 years by age group and individual year, 2006–2019 in uMkhanyakude

| 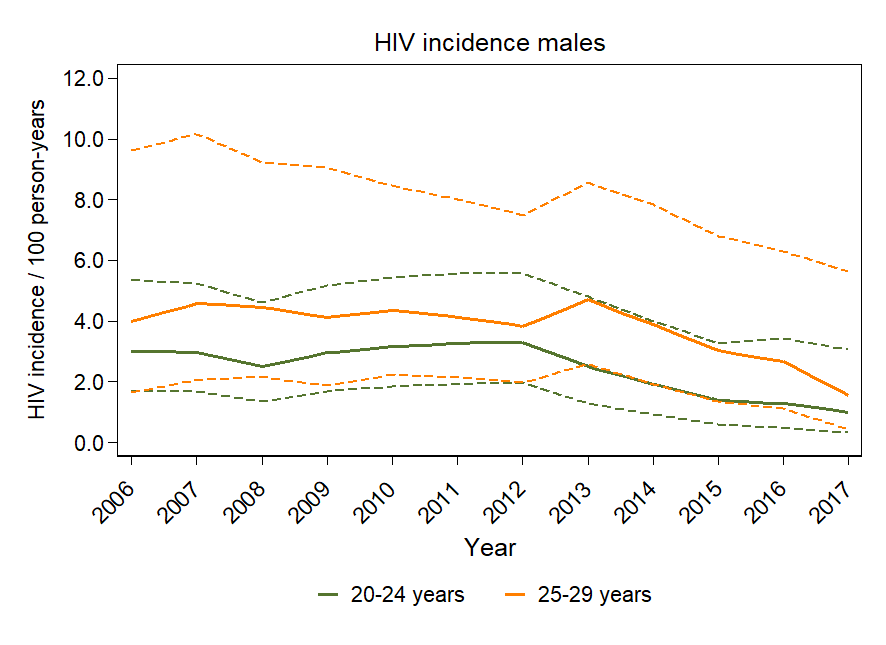**Age group** | **Year** | **New HIV infections** | **Person-years** | **Incidence rate / 100 person-years** | **Rate ratio (95% CI) (reference year 2011)** |
| --- | --- | --- | --- | --- | --- |
| **20–24y** | 2006 | 32 | 932 | 3.05 (1.77 -5.25 ) | 0.95 (0.45 -1.98 ) |
|  | 2007 | 35 | 1007 | 3.08 (1.84 -5.17 ) | 0.96 (0.47 -1.95 ) |
|  | 2008 | 35 | 1096 | 2.59 (1.44 -4.65 ) | 0.81 (0.37 -1.77 ) |
|  | 2009 | 39 | 1136 | 3.02 (1.76 -5.20 ) | 0.94 (0.44 -1.99 ) |
|  | 2010 | 45 | 1237 | 3.23 (1.92 -5.43 ) | 1.01 (0.48 -2.13 ) |
|  | 2011 | 45 | 1267 | 3.21 (1.95 -5.29 ) | 1 |
|  | 2012 | 45 | 1257 | 3.30 (1.97 -5.53 ) | 1.03 (0.48 -2.18 ) |
|  | 2013 | 41 | 1215 | 2.49 (1.36 -4.57 ) | 0.78 (0.35 -1.71 ) |
|  | 2014 | 36 | 1245 | 2.07 (1.08 -4.00 ) | 0.65 (0.28 -1.48 ) |
|  | 2015 | 28 | 1256 | 1.41 (0.63 -3.14 ) | 0.44 (0.17 -1.15 ) |
|  | 2016 | 24 | 1185 | 1.31 (0.53 -3.26 ) | 0.41 (0.15 -1.14 ) |
|  | 2017 | 18 | 1150 | 1.02 (0.39 -2.67 ) | 0.32 (0.10 -0.96 ) |
|  | 2018 | 12 | 1007 | 0.93 (0.34 -2.52 ) | 0.29 (0.09 -0.89 ) |
| **25–29y** | 2006 | 32 | 932 | 4.15 (1.66 -10.33) | 1.01 (0.33 -3.10 ) |
|  | 2007 | 35 | 1007 | 4.43 (2.02 -9.68 ) | 1.08 (0.40 -2.94 ) |
|  | 2008 | 35 | 1096 | 4.36 (2.07 -9.18 ) | 1.07 (0.40 -2.83 ) |
|  | 2009 | 39 | 1136 | 4.11 (2.01 -8.39 ) | 1.00 (0.38 -2.66 ) |
|  | 2010 | 45 | 1237 | 4.37 (2.26 -8.45 ) | 1.07 (0.41 -2.80 ) |
|  | 2011 | 45 | 1267 | 4.09 (2.16 -7.76 ) | 1 |
|  | 2012 | 45 | 1257 | 3.96 (2.12 -7.39 ) | 0.97 (0.38 -2.44 ) |
|  | 2013 | 41 | 1215 | 4.68 (2.70 -8.13 ) | 1.14 (0.49 -2.70 ) |
|  | 2014 | 36 | 1245 | 3.98 (2.19 -7.26 ) | 0.97 (0.40 -2.36 ) |
|  | 2015 | 28 | 1256 | 3.28 (1.69 -6.39 ) | 0.80 (0.32 -2.04 ) |
|  | 2016 | 24 | 1185 | 2.86 (1.35 -6.03 ) | 0.70 (0.26 -1.91 ) |
|  | 2017 | 18 | 1150 | 2.27 (0.94 -5.47 ) | 0.55 (0.19 -1.63 ) |
|  | 2018 | 12 | 1007 | 1.59 (0.54 -4.67 ) | 0.39 (0.11 -1.35 ) |
