# Supplementary material for: Evaluating the impact of DREAMS on HIV incidence among adolescent girls and young women: A population-based cohort study in Kenya and South Africa
Source: PLoS Med. 2021 Oct 25;18(10):e1003837. doi: 10.1371/journal.pmed.1003837 (PMC8880902; doi:10.1371/journal.pmed.1003837)
Supplement: S4 Table — (DOCX) [file pmed.1003837.s005.docx]

**S4 Table.** Incidence of HIV infection among young men in Gem, by age and DREAMS implementation period: *sensitivity analysis without residency gaps*

| **Age group** | **Calendar period** | **New HIV infections** | **Person-years** | **Incidence rate / 100 person-years** | **Age-adjusted rate ratio (95% CI)** |
| --- | --- | --- | --- | --- | --- |
| 20-24 years | 2010-2012 | 5 | 16434 | 0.30 (0.13-0.73) | 1.30 (0.41-4.10) |
|  | 2013-2015 | 7 | 2991 | 0.23 (0.11-0.49) | 1 |
|  | 2016-2019 | 6 | 2725 | 0.22 (0.10-0.49) | 0.94 (0.32-2.80) |
| 25-34 years | 2010-2012 | 22 | 2213 | 0.99 (0.66-1.51) | 1.88 (1.03-3.44) |
|  | 2013-2015 | 20 | 3785 | 0.53 (0.34-0.82) | 1 |
|  | 2016-2019 | 24 | 3195 | 0.75 (0.50-1.12) | 1.42 (0.79-2.57) |
