# Supplementary material for: Evaluating the impact of DREAMS on HIV incidence among adolescent girls and young women: A population-based cohort study in Kenya and South Africa
Source: PLoS Med. 2021 Oct 25;18(10):e1003837. doi: 10.1371/journal.pmed.1003837 (PMC8880902; doi:10.1371/journal.pmed.1003837)
Supplement: Attachment — Submitted filename: Response to reviewers_30.09.21.pdf [file pmed.1003837.s008.pdf]

***

- Are you able to add additional information to the data statement to indicate the folder or files in the two repositories that contain the relevant study data? (Please ensure that the paper adheres to the PLOS Data Availability Policy (see http://journals.plos.org/plosmedicine/s/data-availability), which requires that all data underlying the study's findings be provided in a repository or as Supporting Information. For data residing with a third party, authors are required to provide instructions with contact information for obtaining the data.)

→ *We have now provided the folder or contact information for obtaining the data stored on repositories, as follows:*

  - *The datasets used in this study have been deposited in data repositories and will be available upon reasonable request. Data from South Africa are available from the AHRI data repository (https://data.ahri.org/index.php/home). Requests for the data from Kenya can be made to the KEMRI Scientific and Ethics Review Unit (SERU), at https://www.kemri.org/seru-overview or by contacting SERU at seru@kemri.org*

- Early in the "Methods and findings" subsection of your abstract, please add a sentence, say, to describe the nature and dates of the surveillance surveys.

→ *We have now included this information in the first sentence of the Methods subsection.*

- Again in the abstract, prior to quoting the findings on HIV incidence, please quote some relevant numbers from table 1, which might include the range of survey cohort sizes, the range of proportions contacted, and the range of proportions consented.

→ *We have now included this information in the second paragraph of the Methods and findings.*

- In the abstract, when comparing numbers for HIV incidence, please convert instances of "from X new infections ... to Y" to "X *as compared with* ... Y" or similar.
- Please make similar changes in the Results section (main text).

→ *These edits have now been made in the Results subsection of the abstract and the main text.*

- Please restructure the "Author summary", as the first two points of the "What do these findings mean?" subsection belong in the previous subsection ("What did the researchers do and find?"). Please aim for 2-4 points in each subsection, overall.

→ *We have now moved the first two points of the 'What do these findings mean?' subsection to the previous section, and added two bullet points to the final section*

- In the Methods section (main text), you mention both an a priori analysis plan and study protocol. Are you able to provide one or both documents as attachments? If so, please refer to these in the text.

→ *We have referenced the published study protocol, listed as reference number 5.*

- We suggest avoiding the apostrophe in "DREAMS' introduction" and similar phrases, which will be difficult to make consistent, instead writing "introduction of DREAMS" and the like.

→ *We have now edited this throughout.*

REFERENCES

→ *We have made the following changes to the reference formatting*

- Throughout the text, please style reference call-outs as follows: "... early 2016 [5,6]." (noting the absence of spaces within the square brackets).

- In the reference list, please use the journal name abbreviation "PLoS ONE" consistently.

  Noting reference 2 and others, please add abstract numbers to citations of conference presentations, where available.

- Please update reference 18: it may be necessary to remove this reference if not available as a preprint or "in press".

→ *As this paper is not yet in press or available as a pre-print, we have removed this reference.*

- To enhance the reproducibility of your results, we recommend that you deposit your laboratory protocols in protocols.io, where a protocol can be assigned its own identifier (DOI) such that it can be cited independently in the future.

→ *Thank you for this recommendation. We can include the Kenyan Ministry of Health national guidelines for rapid HIV testing to the reference list, and add supplementary material for the standard operating procedures used in both settings, if the editors advise including them as supplementary material.*

Reviewers

Thank you for this revised manuscript. Overall, this version addresses many of the comments and feedback. However, the reporting of HR throughout remains inconsistent with uncertain language around non-significant results. Could the authors be very clear <u>in the methods (analyses)</u> how they are interpreting results and 95% CI, then use that throughout. Include all p-values (not only some) and then be clear about what is interpreting the "direction" or "magnitude" of a significant or non-significant result. Thank you.

→ *We have edited to improve the consistency of our language, within the Abstract and main document. As noted in our previous response we continue to use the width and lower and upper bounds of the 95% confidence intervals to guide whether there is evidence of change, and do not apply a strict threshold for significance (e.g., Yes/No based on p<0.05). For example, in some cases, we acknowledge that an estimate is 'uncertain' if close to the null value of 1 or the 95% confidence interval is wide.*
