# Supplementary material for: Evaluating the impact of DREAMS on HIV incidence among adolescent girls and young women: A population-based cohort study in Kenya and South Africa
Source: PLoS Med. 2021 Oct 25;18(10):e1003837. doi: 10.1371/journal.pmed.1003837 (PMC8880902; doi:10.1371/journal.pmed.1003837)
Supplement: Attachment — Submitted filename: PMEDICINE-D-20-06166-R1.docx [file pmed.1003837.s006.docx]

Thank you for the opportunity to review this manuscript. This is an important and well written manuscript assessing the impact of the DREAMS programme in two sub-Saharan African settings (one in South Africa and one in Kenya) in terms of reducing HIV incidence among young women and men. The piece is strong and utilizes data where serosurveys were available for periods before and after DREAMS implementation. There were many sensitivity analyses/different assessments of time, which could cause concern around multiple hypothesis testing, yet overall the findings were robust: HIV incidence declined over time, but the trend started and did not accelerate during the DREAMS implementation period. There were a few areas that the authors could have provided greater justification for in terms of differences in analytic approach across settings, but overall my largest concern/thought was why didn’t the authors use an interrupted time series analysis. It seems like much of what they were trying to do would have been better served by this method? Also, there was a non-insignificant (though understandable) loss to follow-up over time and how those with follow-up incidence data compared to those without within and across time periods was not explored and potentially could be handled to ensure the robustness of the results (for example using inverse probability weighting). Overall, this is important but considerations of these analytic approaches would strengthen the robustness of the findings.

Abstract: ---

Introduction:

The introduction was well written – no comments

Methods:

For the power calculation, it is disorienting (and feels almost deceptive) to give such different power calculations across the two settings. Unless the DREAMS targets were different in each setting (if so this should be clarified), having a 30% reduction and 90% power in one place and 45% reduction and 80% power (or in parentheses 40% reduction & 70% power) is confusing. It seems best to determine the threshold that you think critical- 40% was the DREAMS target so might be logical and be consistent with the difference aimed to detect across settings.

Similarly, why were the number of imputations different (n=100 vs. n=250) across sites? If there is a reason please provide, otherwise it would seem that these should be congruous.

How was LTFU handled? The authors note that individuals were included if they had 2+ visits, but for those that were not out-migrations (and censored temporarily or permanently), how was follow-up handled. Inverse probability weighting to account for whom follow-up data was and was not available may be used here and could be important depending on the extent to which follow-up occurred.

One of the assumptions of Poisson regression is a steady incidence rate within time periods. Was this assumption assessed? Did the authors consider an interrupted-time series as a potential approach to assess changes in trends over time as related to the DREAMS intervention implementation? These types of quasi-experimental designs are increasingly used.

Please provide a justification within the methods for why different age bands were considered in relation to men across the two settings.

RESULTS

Please describe how those with and without follow-up in both sites were different/similar.

Also, why were the participants contacted so much lower in Gem (34%) vs. uMkhanyakude (85%)?

There is no accounting for these issues of contact and follow-up and at least for follow-up methods to account for this (at least in part) could be applied.

Per Table 1b, what happened in 2019 that made only 0.7% of testing results available for those eligible for the HIV incidence cohort?

Table 4 – unclear why comparison groups (categories for which incidence is reported) across years differ by age band.

Discussion

The authors’ point in the discussion that interventions may not meet the needs of those at highest risk (such as women engaged in sex work or transactional sex) is important and perhaps could be expounded upon.

The authors should mention as a limitation (and ideally attempt to address) the potential impact of LTFU over intervals on their results.
