# Supplementary material for: Evaluating the impact of DREAMS on HIV incidence among adolescent girls and young women: A population-based cohort study in Kenya and South Africa
Source: PLoS Med. 2021 Oct 25;18(10):e1003837. doi: 10.1371/journal.pmed.1003837 (PMC8880902; doi:10.1371/journal.pmed.1003837)
Supplement: Attachment — Submitted filename: Response to reviewers_31.03.21.docx [file pmed.1003837.s007.docx]

Manuscript: PMEDICINE-D-20-06166R1

“Evaluating the impact of DREAMS on HIV incidence among adolescent girls and young women in large population-based cohorts in Kenya and South Africa”

***** RESPONSE to Reviewers**

***** Requests from the editors**

Please add a colon followed by a study descriptor (e.g., "...: A before-and-after study") to the title. Please remove the word "large".

As you study the situation in young men in addition to other groups, we suggest amending the title as appropriate.

- *We have now changed the title from:*

*“Evaluating the impact of DREAMS on HIV incidence among adolescent girls and young women in large population-based cohorts in Kenya and South Africa” to:*

- *“Evaluating the impact of DREAMS on HIV incidence among adolescent girls and young women: a population-based cohort study in Kenya and South Africa”*

As you study the situation in young men in addition to other groups, we suggest amending the title as appropriate.

- *We have kept the focus of the title on adolescent girls and young women as this was the target group for the intervention (DREAMS). The findings on young men were conducted for context and to understand if they could have impacted on young women.*

Please add a few words to your abstract to describe what DREAMS consists of - we suggest early in the "Methods and findings" subsection, along with the program dates.

- *We have now expanded our description of DREAMS in the “Background” subsection of the Abstract, as follows: “DREAMS promotes a combination package of evidence-based interventions to reduce individual, family, partner, and community-based drivers of young women’s heightened HIV risk.”*
- *We have also included program dates in the “Methods and findings” subsection.*

Please avoid "38% lower" and the like where an aRR is quoted.

- *We have now removed percentages where an aRR is presented in the Abstract.*

Please add a new final sentence to the "Methods and findings" subsection of your abstract, beginning "Study limitations include ..." or similar and quoting 2-3 of the study's main limitations.

- *We have now added: “Study limitations include low study power in Kenya and the introduction of other interventions such as universal treatment for HIV during the study period.”*

You mention an a priori analysis plan in the Methods section. Please attach the plan as a supplementary file if available, referred to in the main text. Please highlight analyses that were not prespecified.

- *We have included a reference to the study protocol, where the a priori analysis is described. [Discussion ¶6]*
- *Analyses that were not pre-specified are labelled as ‘a posteriori’.*

Throughout the text, please format reference call-outs as follows: "... HIV risk [12,13]." (noting the **absence of spaces** within the square brackets).

- *We have tried to apply these formatting changes using the Vancouver style, however, our reference manager (EndNote X9) uses round parentheses () rather than square brackets [], to offset the reference numbers. It also automatically places a space in between the reference numbers. May we ask your assistance to change this formatting? The use of punctuation and plain text and first 6 author names has now been corrected.*

In the reference list, please **convert all italics into plain text**. Where appropriate, please list **6 author names followed by "et al.".**

Please adapt the **use of punctuation** in lists of author names to journal style (i.e., "Surname Initial(s), Surname Initials, ..., et al.").

Please **remove information on funding from the end of the text**. In the event of publication, this information will appear in the article metadata via entries in the submission form.

- *Information on funding has now been deleted from the text.*

Please add a completed checklist for the most appropriate reporting guideline, e.g., STROBE, as a supplementary file, labelled "**S1_STROBE_Checklist**" or similar and referred to as such in the Methods section. In the checklist, please refer to individual items by section (e.g., "Methods") and paragraph number rather than by line or page numbers, as the latter generally change in the event of publication.

- *We have now completed and submitted a STROBE Checklist for Cohort studies, and attached as a supplementary file (S1) and referred to in the Methods ¶1.*

***** Comments from the reviewers**

***** Reviewer #1**

This is a useful study on the impact of DREAMS on HIV incidence among adolescent girls and young women in two communities in Kenya and South Africa. The statistical methods and analyses are relatively straightforward and mostly adequate. However, there are some major issues needing attention.

1) For the sampling of the cohort in uMkhanyakude, South Africa, the consent rates are generally low (40%-60%); for the Gem data in Kenya, the contact rate and also the HIV results rate are both very low (<50%).

- *We appreciate the positive comments by this reviewer, and the suggestion to discuss participation rates in more detail. We have now done so within the Discussion ¶9 (Strengths & Limitations), where we explain the following:*
  - *“With annual rounds of sero-surveillance in South Africa, participation rates in any particular year were relatively low (for example, due to absence from home or study fatigue), however, cumulative rates of participation among eligible individuals increased with time and recruitment opportunities. Overall, about 75% of eligible AGYW contributed person-time during follow-up, which is relatively high for population-based surveillance.”*
  - *“In Kenya, the proportion contacted and offered an HIV test was lower, since surveys were done primarily as service provision (home-based testing to link HIV-infected individuals to care), and those with a known HIV positive status did not require a new test. However, the approach to reaching people has remained consistent over time and the population reached is comparable over time. [Borgdorff 2019] With less frequent sero-surveillance in this setting compared to South Africa, and high mobility among young adults, there was less opportunity for repeat testing and thus the proportion who contributed person-time to our analyses was lower.”*
  - *We have no evidence that this introduced significant bias (see further detail on this below), however, it limited the sample size and study power to detect a difference of 40% in Gem (which we have addressed in the Discussion).*

For such high missing rates in a population study, what's the likely impact on the true estimate of the trends in reduction of HIV rates? Underestimate or overestimate? This needs to be carefully discussed in the limations. For such a high missing rate, the reliability and believeability of results are subject to scrutiny

- *We have now included text and references to address this point in the Discussion ¶9, as follows:*
  - *“While repeated HIV testing via population-based surveillance is considered by many to be the gold standard for measuring HIV incidence trends [Vandormael 2019], such studies are challenged by the systematic exclusion of some sub-groups due to non-contact or refusal. [Larmarange 2015]* *The consequence is most likely that absolute levels of HIV incidence in this study and others may be under-estimated, because more mobile groups are disproportionately missed. However, as noted above, in each calendar year of follow-up in uMkhanyakude a high proportion of eligible AGYW contributed to the analysis, and the proportion contributing to the analysis was fairly consistent over the study period. Also, in previous analyses of participation dynamics in the South Africa surveillance site, the HIV testing rate did not differ substantially by age or sex over time and the demographic composition (age and sex) of the HIV-negative testers and the HIV cohort remained stable over time.” [Larmarange 2015; Vandormael 2019]*
- *Also, we have now explored differences between those who did and did not contribute data (person-time) to the HIV incidence estimates. Please see response to Reviewer 2 below.*

2) Study design. This is a before and after cohort design to exam the impact after introduction of the complex intervention of DREAMS on reducing HIV rates over time. However, how can we be certain that the observed reduction in HIV rates is due to DREAMS only? In other words, we need controls/bench marks to show that the reduction in HIV rates is truely because of DREAMS. Do authors have the national HIV rates for South Afric and Kenya over the same time periods? If they are also in decline (e.g. due to national HIV prevention campaign), then there could be contamination effect that needs to be carefully addressed. Or, the HIV rates over time in the neighbouring areas of these two communities? The observed big reduction in HIV rates in young men in the same period doesn't help the arguement. It means the rates declined without DREAMS for young men. Intuitively one would think the rates in young women might decline anyway without DREAMS in the same period. Then, the question is what is the true impact of DREAMS if any. Without addressing all these questions, one can not confirm the impact of DREAMS with confidence.

- *Thank you for this query. We agree that the observed declines in HIV incidence cannot be attributed to DREAMS. We have checked to ensure that this conclusion is clear, for example, in these sections:*
- *“We conclude that the declining trend began several years before DREAMS’ introduction and continued, but did not accelerate, during DREAMS’ first three years of implementation. [Discussion ¶1]*
- *“the declines pre-dated DREAMS introduction in both settings, and thus cannot be attributed to the initial years of DREAMS interventions. [Conclusion, final ¶]*
- *The findings of HIV incidence declines among young men, which preceded those observed among young women, are an important part of the picture and explanation. We conclude that the observed HIV declines among AGYW “are likely due to time trends in population- and partner-level risks which began before DREAMS and continued afterwards.” [Discussion ¶3 and conclusion of the abstract and discussion]*

***** Reviewer #2**

Thank you for the opportunity to review this manuscript. This is an important and well written manuscript assessing the impact of the DREAMS programme in two sub-Saharan African settings (one in South Africa and one in Kenya) in terms of reducing HIV incidence among young women and men. The piece is strong and utilizes data where serosurveys were available for periods before and after DREAMS implementation. There were many sensitivity analyses/different assessments of time, which could cause concern around multiple hypothesis testing, yet overall the findings were robust: HIV incidence declined over time, but the trend started and did not accelerate during the DREAMS implementation period. There were a few areas that the authors could have provided greater justification for in terms of differences in analytic approach across settings, but overall my largest concern/thought was why didn't the authors use an interrupted time series analysis. It seems like much of what they were trying to do would have been better served by this method? Also, there was a non-insignificant (though understandable) loss to follow-up over time and how those with follow-up incidence data compared to those without within and across time periods was not explored and potentially could be handled to ensure the robustness of the results (for example using inverse probability weighting). Overall, this is important but considerations of these analytic approaches would strengthen the robustness of the findings.

- *We are grateful for this careful review and the positive and constructive comments are well received. We have responded to each of the questions and suggestions below.*

Abstract: ---
Introduction:
The introduction was well written - no comments
Methods:
For the power calculation, it is disorienting (and feels almost deceptive) to give such different power calculations across the two settings. Unless the DREAMS targets were different in each setting (if so this should be clarified), having a 30% reduction and 90% power in one place and 45% reduction and 80% power (or in parentheses 40% reduction & 70% power) is confusing. It seems best to determine the threshold that you think critical- 40% was the DREAMS target so might be logical and be consistent with the difference aimed to detect across settings.

- *We agree with this feedback and have now edited this section using 40% power as the threshold in both settings, as follows:*
- *“In South Africa, where pre-DREAMS annual HIV incidence was estimated to be 6% among 15-24 year-old AGYW, we estimated there would be at least 3000 person-years of follow-up after DREAMS roll-out and that study power was 95% to show a 40% reduction in HIV incidence. In Kenya, where pre-DREAMS HIV incidence was much lower, at 0.7% among AGYW aged 15-24 years, we estimated that with 9000 person-years of follow-up, study power was 70% to show a 40% reduction in HIV incidence.” [Methods ¶3]*

Similarly, why were the number of imputations different (n=100 vs. n=250) across sites? If there is a reason please provide, otherwise it would seem that these should be congruous.

- *We felt that a minimum of 100 imputations was adequate and we have now stated this within the Methods. [Methods ¶5] Conducting 250 imputations with the South Africa data allowed for greater certainty with the more detailed analysis of HIV sero-conversions by calendar year and post hoc calendar periods (presented in Table 3 and 4, respectively).*

How was LTFU handled? The authors note that individuals were included if they had 2+ visits, but for those that were not out-migrations (and censored temporarily or permanently), how was follow-up handled. Inverse probability weighting to account for whom follow-up data was and was not available may be used here and could be important depending on the extent to which follow-up occurred.

- *As an open cohort, we sought population-level change by age group, sex and time, and the analyses were adjusted for age in quite fine age groups (5 years)*. *The proportion of eligible AGYW who contributed data to HIV incidence estimates is relatively consistent over time, and previous research shows that the HIV testing rate did not differ substantially by age or sex over time and the demographic composition (age and sex) of the HIV-negative testers and the HIV cohort remained stable over time [Larmarange 2015]. This is now noted in the Discussion ¶9, Strengths & Limitations.*
- *However, to explore the possibility that differential attrition by individual year of age altered the underlying population, we have now added the following to the Methods: “We compared the age distribution of the eligible AGYW who did and did not contribute person-years of follow-up to the HIV incidence analyses in each calendar year (those with and without repeat HIV tests during or after the calendar year being considered).” [Methods ¶4]*
- *And, within the Results ¶1, we now add: “For each calendar year of follow-up, AGYW who contributed person-years to the analysis were on average slightly older than those who did not contribute; the differences in mean age were small (<1 year) and changed little over the study period,” with a reference to the new Table S1 with these results. The slightly older age is most likely due to the greater opportunity to re-test with time since first eligibility for HIV testing. This pattern is consistent across the calendar years of our study, and combined with the difference in mean age being small, suggests that analysis of trends over time were not biased by repeat and non-repeat testers differing by age.*
- *Also, in earlier analyses based on data from the same HIV surveillance systems, including risk factor analyses: authors in South Africa found that sensitivity analyses with inverse probability of survey weights strongly supported the robustness of the estimated trends in HIV incidence [Vandormael 2019]; and authors in Kenya found that attrition was not associated with HIV prevalence [Borgdorff 2019].*

One of the assumptions of Poisson regression is a steady incidence rate within time periods. Was this assumption assessed?

- *The analysis in South Africa estimated yearly HIV incidence. In the Kenyan setting, this was also the case in the post-DREAMS calendar period (with annual surveys between 2016-2019). It was not possible to have finer calendar time periods pre-DREAMS in Kenya given the sero-survey intervals, so the HIV estimates are over an average period of ~2-3 years. It is true that there may be a decline within that multi-year calendar period, however, our assumption is that change in HIV incidence will not be rapid.*

Did the authors consider an interrupted-time series as a potential approach to assess changes in trends over time as related to the DREAMS intervention implementation? These types of quasi-experimental designs are increasingly used.

- *With regard to interrupted time series (ITS) analysis, our post-hoc analysis of time trends in uMkhanyakude fit within the framework of ITS, and our a priori analysis shares a key feature of ITS. Both analyses defined a key time point, namely the year that DREAMS was first rolled out. We then framed the question as whether the HIV incidence rate was different before and after this time point. Our post-hoc analysis fits within the framework of ITS, asking the question of whether the slope of HIV incidence decline changed after DREAMS roll-out; specifically, this analysis is within the framework of Example 1 in the paper by Kontopantelis et al, BMJ 2015 (“Regression based quasi-experimental approach when randomisation is not an option: interrupted time series analysis”). The analysis is not ‘interrupted’ per se since a sudden change (a ‘discontinuity’) would not have been expected. Rather, a shift in the trajectory of change that would build up over time. We assumed that any change would be gradual as DREAMS took time to roll-out and implement all components at scale.*
- *We have now added this point to the Methods, with: “This approach fits within the framework of an interrupted time series analysis as it identifies a key time point, and then asks whether a time trend alters the trajectory (slope) after that time point.” [Methods ¶7]*

Please provide a justification within the methods for why different age bands were considered in relation to men across the two settings.

- *In Methods ¶8, we have now emphasised that the age ranges for men are based on our earlier research showing that the typical age range for sexual partners of AGYW differed by setting, i.e., 20-29 years in the South Africa setting and 20-34 years in the Kenya setting. [Reference: Doyle 2019, PLOS One 2018]*

RESULTS

Please describe how those with and without follow-up in both sites were different/similar.

- *This has now been added to the Results ¶1 and new Supplemental Table S1.*

Also, why were the participants contacted so much lower in Gem (34%) vs. uMkhanyakude (85%)? There is no accounting for these issues of contact and follow-up and at least for follow-up methods to account for this (at least in part) could be applied.

- *Please see our response to Reviewer #1, point (1) above, including the new text added to explain participation rates and their implications (Discussion ¶9, Strengths & Limitations). We have responded to the point about LTFU in the section above.*

Per Table 1b, what happened in 2019 that made only 0.7% of testing results available for those eligible for the HIV incidence cohort?

- *Few participants in the 2019 round in Gem contributed to the HIV incidence estimates because additional serological data (collected after 2019) is needed to establish whether sero-conversion occurred in 2019. This was not the case in South Africa where we censored the analysis at the end of 2018, and used 2019 serological data to estimate HIV incidence in 2018 (and thus improved the accuracy of the tail-end of trend estimates). We explain in the Methods ¶6 that this difference in approach is due to the fact that DREAMS investments stopped in South Africa by late 2018 and continued in Kenya. We also state in the Discussion ¶10 that “longer-term follow up, with the benefit of additional serological data collected after 2019, would increase the accuracy of the ‘tail-end’ of our estimates.”*

Table 4 - unclear why comparison groups (categories for which incidence is reported) across years differ by age band.

- *We chose the time periods informed by the year in which HIV incidence first began to decline, for 15-19 year olds and 20-24 year olds, based on results in Table 3 and Figure 1A, and this differed by age group. We have now added a footnote to Table 4 to make this more clear.*

Discussion
The authors' point in the discussion that interventions may not meet the needs of those at highest risk (such as women engaged in sex work or transactional sex) is important and perhaps could be expounded upon.

- *Our related research has found that young women who sell sex (YWSS) face stigma by implementing organisations that require more training and experience to address the unique needs of YWSS. We have now mentioned these issues of stigma and inclusiveness in this part of the Discussion ¶5. In the same paragraph, we have added findings from our earlier analysis of DREAMS participants, showing that those who may be at most immediate sexual risk were less likely to be invited to DREAMS.*

  The authors should mention as a limitation (and ideally attempt to address) the potential impact of LTFU over intervals on their results.
- *We have now discussed this issue in a new paragraph ¶9 within the Discussion, drawing on the responses and additional analyses described above.*

***** Reviewer #3**

Thank you for the opportunity to review this article - it is such important research considering the need to prioritize funding for programming in the post-COVID-19 era.

- *Thank you for this positive response, and the important questions raised below to strengthen the manuscript.*

My feedback is two-fold:

big picture:
* the authors do not touch on this but it is a critical question to evaluating impact/ effectiveness: quality of DREAMS implementation. While there is an acknowledgement of the timing of introducing DREAMS (start and end) and how set up may take time, a point in the discussion on how quality and reach of DREAMS may have shaped the results documented.

- *We have now provided additional details about quality and reach of DREAMS interventions in the second, third and fifth paragraphs of the Discussion.*

* where to next? It would be helpful for authors to reflect on the implications of their really impressive research in two key ways: (1) integrating and enhancing data collection in implementation of large-scale programmes - costs, skills, timelines, (2) methodologies of measuring and evaluating impact in scaled up programming. i.e. should there always be a lagged approach to expecting, documenting and analysing for impact?

- *We agree that time is needed for program implementation to be scaled-up as intended (in this case, to district level and to a considerable proportion of the population) and that for many outcomes time is also needed before an impact is achieved. The process & timing of change varies by outcome of interest, and outcomes like HIV incidence, empowerment, agency and gender inequities are expected to change gradually. We argue in the Discussion and Conclusion that DREAMS’ primary goal, to impact HIV incidence by 40% with real-world implementation of a complex intervention, is likely to require more than two years. Analyses of other outcomes which are quicker to change, such as uptake of services like HIV testing and treatment, use of condoms and PrEP, can be done more quickly as and after interventions are introduced. Regular updates between the authors (as independent evaluators) and the funders and implementers of DREAMS were useful for timely response and adaptation based on emerging insights throughout the implementation period.*
- *We have now added to the Discussion ¶6: “DREAMS is a very large investment and direct measurement of HIV incidence at population level, at least in some settings, is warranted to evaluate whether DREAMS achieved its target for HIV incidence reduction. Capitalising on existing research platforms was an efficient way to evaluate this aim.”*

detailed questions/ items to clarify:
* power calculations - while it makes sense that they vary by site since data collection designs were different, it is unclear why the SA goal was 90% power to detect 30% while Kenya 80% to detect 45%? This is also a bit confusing because in SA the PY included were then much larger than the power calculation.

- *We agree with this feedback. Please see our response to Reviewer 2 above, and the text in the Methods now edited with 40% power as the threshold in both settings. [Methods, ¶3]*

* air-time - please give amount and who/ where it was sent to (i.e. young adolescent or their caregiver's phone?)

- *We have now specified that compensation of 10 Rand ($0.70) in mobile phone airtime was provided to the 15-24 year-old living in the household. [Methods, ¶3]*

* p-values, please add these throughout to make it easy to follow your interpretation - some of the narrative around the reported values sometimes speaks about impact and at times does seem close to a non-significant. Could the authors please add all p-values and be clear about significance vs interpretation of trends, even when not significant throughout the manuscript. This is quite important because in page 10 the authors report: "but there was a decline among the 20-24 year olds (aRR=0.69, 95%CI 0.53-2.18), albeit with considerable uncertainty around these estimates." but the discussion then speaks to "There is one exception to this finding: among young women aged 20-24 years in Gem, HIV incidence remained stable prior to DREAMS introduction (at circa 1% between 2010-2015), and declined by 31% during the DREAMS implementation period 2016-2019 but with considerable uncertainty around this estimate." but as I understand the data, this reduction is non significant and with a wide confidence interval. To help all of us reading the manuscript, it would be helpful to set the parameters for defining reduction and the types of reduction (significant/ certain; uncertain) in the methods.

- *This is helpful feedback, and we have now removed the phrase “There is one exception to this finding” and added “statistical evidence (a wide confidence interval, indicating the data were compatible with a rate ratio of 1), shows this may be a chance finding.” [Discussion ¶2] We have used this criteria, i.e., the width and lower and upper bounds of the 95% confidence intervals, throughout the manuscript to guide whether we have evidence of impact along a continuum of strong, weak, or no evidence (as opposed to a strict threshold for significance Yes/No based on p=0.05).*
- *We have also edited this finding in the Results ¶6 to avoid ambiguity, by replacing “there was a decline” with “there was no evidence of a decline in HIV incidence during the DREAMS implementation period” [in either age group].*

* in the discussion the authors speak to positive causal impact on related outcomes - but the two references are quite broad and dont clarify methods, etc. Could they include some more detail in the discussion until publications are available with the data?

- *We have now elaborated within the Discussion ¶2: “As part of the same evaluation, we established and followed nested closed cohorts of AGYW, randomly sampled from the population platform and followed over two years, to measure causal impacts on individual-level outcomes. In Gem, we observed a reduction in lifetime number of sexual partners and condomless sex among DREAMS beneficiaries compared with non-beneficiaries, and evidence of other changes along a causal pathway between DREAMS and HIV risk, such as social support.”*
- *We have also added references to conference presentations with further details on the methodology and findings.*

It is really great to see these findings - all of us in the HIV prevention community have been looking forward to this data and learning from DREAMS.
